# Efficacy and safety of moxibustion for benign prostatic hyperplasia: A protocol of systematic review and meta-analysis

**DOI:** 10.1097/MD.0000000000028437

**Published:** 2021-12-23

**Authors:** Jiaze Wang, Tingting Deng, Hao Sun, Xiaolu Sun, Yuwei You, Ying Wang, Qi Xun, Yuxia Ma

**Affiliations:** a Shandong University of Traditional Chinese Medicine, Jinan, Shandong, China; b Affiliated Hospital of Shandong University of Traditional Chinese Medicine, Jinan, Shandong, China.

**Keywords:** benign prostatic hyperplasia, meta-analysis, moxibustion, protocol, systematic review

## Abstract

**Background:**

Benign prostatic hyperplasia (BPH) is a non-malignant enlargement of the prostate gland that is common in older men. The clinical manifestations of BPH are frequent urination, urgency, incomplete dribbling of urine, and urinary retention. Moxibustion, as a convenient, safe and effective method, has been widely applied in the clinical treatment of BPH. The study aim to assess the efficacy and safety of moxibustion for BPH.

**Methods:**

The following electronic databases will be searched regardless of language and publication status: Pubmed, MEDLINE, EMBASE, China Biomedical Database, China National Knowledge Infrastructure, VIP Database, and Wanfang Database, to select studies that meet the requirements. The study will consist of a prospective randomised controlled clinical trials (RCTs) of moxibustion in the treatment of BPH, language of publication does not have barrier of blinding or restrictions, adverse events will be assessed and reported for safety assessment. Two reviewers will independently conduct and screen all included studies and the meta-analysis will be performed with RevMan V5.3.

**Results:**

The study will provide a high-quality convincing assessment of efficacy and safety of moxibustion for BPH.

**Conclusion:**

The conclusion of this study will provide the latest evidence for judging whether moxibustion is effective and safe in the treatment of BPH.

**Trial registration number:**

INPLASY2021120021.

## Abstract 2

Supplemental Digital Content is available in the text

## 1 Introduction

Benign prostatic hyperplasia (BPH) is a common disease in older male group, with an increasing incidence along with age.^[1]^ Although the prevalence is only 8% between the ages of 40 and 50, it increases to 50% between the ages of 51 and 60, and to 83% by the age of 80.^[2,3]^ The clinical manifestations of BPH are frequent urination, urgency, incomplete dribbling of urine and urinary retention,^[1]^ which are collectively referred to as lower urinary tract symptoms, and these symptoms seriously affect patient's sleep, daily social interactions and further lead to depressive symptoms.^[4]^ The main pathological change occurring within the prostate hyperplastic tissue is stromal cell proliferation.^[5,6]^ Androgens are the most important factor influencing the development of BPH,^[7]^ but they do not directly cause proliferative changes in prostate cells, but indirectly trigger proliferative changes in prostate tissue cells by stimulating the secretion of relevant growth factors from prostate stroma and epithelial cells.^[8,9]^ However, there is no clear mechanism for the development of BPH, which may also involve chronic inflammation of the prostate, vitamin D deficiency and other theories. Medication and surgery are common treatments for patients with BPH. Initial treatment for BPH is medication, usually 5-a-reductase inhibitors or alpha-blockers, in addition to other types of therapeutic agents such as M-receptor antagonists^[10]^ and herbal preparations. If pharmacological treatment fails, surgical treatment (transurethral resection of the prostate or open surgery) is used for treatment.^[11]^ For patients with BPH with a prostate volume (PV) of 30 to 80 mL, transurethral resection of the prostate (TURP) is considered the gold standard of treatment and is also indicated for those with a prostate volume >80 mL.^[12,13]^ Although this treatment is effective, it is highly invasive to the operator, has a long postoperative recovery time, and patients may experience various complications associated with TURP.^[14,15]^ Therefore, it is particularly important to choose an alternative therapy with good efficacy and few complications. Moxibustion has been found to have therapeutic effects on a variety of diseases through thermal stimulation.^[16,17]^ It has been found that moxibustion can, through its warm stimulating effect, cause vasodilatation, enhance blood flow dynamics, and lower vascular resistance, thus effectively reducing blood viscosity and improving microcirculatory disorders and blood rheology abnormalities in patients with BPH.^[18–20]^ Up-to-date, there is no evidence of systematic evaluation of moxibustion for BPH. Therefore, in order to obtain convincing evidence for clinical practice, we will systematically review published randomized controlled trials and compare the efficacy and safety of moxibustion with pharmacological/non-pharmacological interventions for BPH.

## 2 Methods and analysis

### 2.1 Study registration

The systematic review has been registered in INPLASY (INPLASY2021120021) and will be conducted according to the preferred reporting items of the system evaluation and the Meta-analysis protocols 2015 statement. This protocol has been checked with Preferred Reporting Items for Systematic review and Meta-Analysis Protocols (PRISMA-P) checklist.

### 2.2 Inclusion criteria for study selection

#### 2.2.1 Types of studies

**Figure 1:**
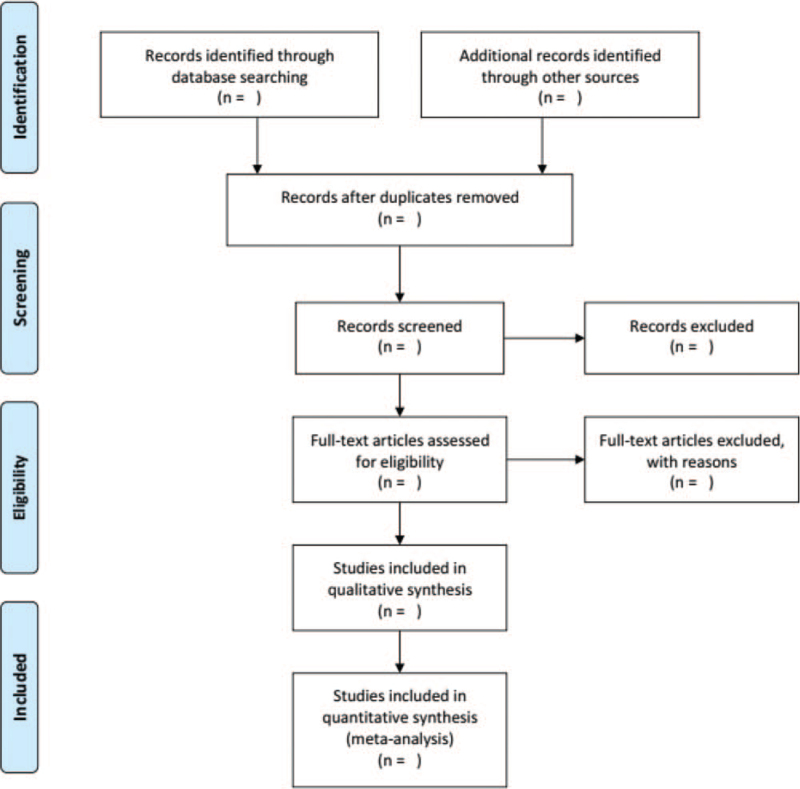
PRISMA flow diagram.

The study will consist of a prospective randomised controlled clinical trials (RCTs) of moxibustion in the treatment of BPH, language of publication does not have barrier of blinding or restrictions. A PRISMA flowchart will be drawn to illustrate the study selection process (Fig. 1).

#### 2.2.2 Participants

Male patients with symptomatic BPH will be participants with no restrictions on age, duration, race, disease duration, or disease severity. These patients appeared with higher than normal urinary symptom scores including the Boya sky score^[21]^ and the International Prostate Symptom Score (IPSS).^[22]^

#### 2.2.3 Types of interventions

The treatment group will include any type of moxibustion therapy such as direct moxibustion, indirect moxibustion, thermosensitive moxibustion, natural moxibustion, medicinal moxibustion as part of the individual treatment or working along with other interventions. Meanwhile the control group will be other types of interventions including medication, surgery, and other conventional treatments.

#### 2.2.4 Types of outcome measures

The primary outcome will be changes in urinary symptoms, including the Boya sky score^[21]^ and the IPSS.^[22]^ Secondary outcomes will include quality of life scores; urodynamic indicators, change in maximum urine flow (in mL/s); mean urine flow (in mL/s) and residual urine volume (in mL), change in prostate size (in cc) and adverse events like dizziness, nausea, and vomiting.

#### 2.2.5 Exclusion criteria

Researches with the following characteristics will be excluded: non-randomised controlled trials; studies with interventions that do not meet the requirements of Ayurveda; studies involving patients with non-BPH; case reports, animal studies, reviews, conference papers, or reports with incomplete significant data that have not been responded to by the appropriate author.

### 2.3 Search strategy

The following electronic databases will be searched regardless of language and publication status: Pubmed, MEDLINE, EMBASE, China Biomedical Database, China National Knowledge Infrastructure, VIP Database, and Wanfang Database. Prospero, Clinic Trials.gov, and Google Scholar will be used to select systematic reviews or ongoing or completed clinical trials. Meanwhile, papers and bibliographies for inclusion in the trials will also be reviewed. The search strategy in Appendix 1, http://links.lww.com/MD2/A795.

### 2.4 Data collection and analysis

#### 2.4.1 Selection of studies

Two evaluators independently screened the literature according to inclusion and exclusion criteria. Abstract and main content of the literature that met the screening criteria will be selected after initial analysis. Final selection will be conducted on the basis of the original text of the selected literature. The 3rd evaluator will come involve when there is disagreement. Extracted information includes first author and year of publication, country, number of patient cases, interventions, follow-up time, and outcome indicators. A PRISMA flow chart will be drawn to illustrate the study selection process (Fig. 1).

#### 2.4.2 Data extraction and management

Authors will be responsible for data extraction and management according to the search strategy, including first author and year of publication, country, number of patients, interventions, follow-up time, and outcome indicators, adverse events will be assessed and reported for safety assessment.

### 2.5 Data analysis

#### 2.5.1 Statistical analysis

Meta-analysis was conducted using the RevMan 5.3 software provided by the Cochrane Collaboration Network. Relative risk (RR) was used for dichotomous variables. For continuous variables, mean squared deviation (MD) was chosen as their effect indicator, and standardised mean squared deviation (SMD) was applied when the units of measurement of the observed indicators differed, with effect sizes expressed as 95% confidence intervals (CI). A narrative synthesis will be provided when meta-analysis of all or some of the expected data from included studies is not possible.

#### 2.5.2 Dealing with missing data

If there is insufficient or missing experiment data, we will attempt to contact the appropriate author(s) through a variety of methods. In cases where authors are unable to provide missing data or cannot be contacted, analyses will be conducted based on the available data and the potential impact of missing data will be discussed as well.

#### 2.5.3 Risk assessment of included studies

The quality of the included literature was assessed using the risk of bias assessment tool recommended in the Cochrane Systematic Assessor's Handbook 5.1.0. Specific methods included: randomization methods, allocation concealment, blinding of subjects and investigators, blinding for outcome assessment, completeness of outcome data, selective reporting of results, and other sources of bias, each of which was categorised as low risk of bias, high risk of bias, and unclear.

#### 2.5.4 Assessment of heterogeneity

The degree of heterogeneity was evaluated against *X*^2^ value, *P* value of heterogeneity test and *I*^2^ index. When *P* > .10 and *I*^2^ < 50%, there was no statistical heterogeneity among studies, and the fixed-effect model was used for meta-analysis. If there was heterogeneity among studies (*P* ≤ .10 or *I*^2^ ≥ 50%), the source of heterogeneity was analyzed first. When there was no obvious clinical heterogeneity and heterogeneity could not be found, the random-effect model was used for meta-analysis.^[23]^

#### 2.5.5 Assessment of reporting bias

Potential reporting bias will be investigated using funnel plots and linear regression methods to measure asymmetry in funnel plot.^[24]^

#### 2.5.6 Sensitivity analysis

Sensitivity analyses are conducted to explore the impact of methodological quality and sample size on the robustness of the review findings, based on the effects of sample size, methodological quality and missing data. The meta-analysis will be repeated after excluding studies with a high risk of bias and outliers that are numerically distant from the rest of the data. The sensitivity analyses will be reported with the summary tables and the review conclusions will relate to comparisons between the two meta-analysis.

#### 2.5.7 Subgroup analysis

If heterogeneity is assessed as significant (*I*^2^ ≥ 50%) and sufficient trials are included, subgroup analyses will be conducted subgroup analyses to explore potential sources of heterogeneity depending on the intervention, control, and outcome measure.

#### 2.5.8 Grading the quality of evidence

Four levels: high, moderate, low, or very low will assess of quality of evidence based on recommended guidelines for assessment, development, and evaluation.^[25]^ The quality of evidence is usually judged on the basis of bias, inconsistency, indirectness, inaccuracy, and risk of publication bias.

#### 2.5.9 Ethics and dissemination

Ethical approval is not required as our research will not include animals or individuals and will not be linked to personal patient data. The findings of this article will eventually be published in a conference or peer-reviewed journal.

## 3 Discussion

Traditional Chinese medicine ascribed BPH to “Retention of urine” or “Stranguria.” On the basis of traditional Chinese medicine theory, deficiency of kidney-essence leading to renal deficiency and blood stasis, is the root cause of its occurrence. Numerous clinical studies have confirmed that moxibustion is effective in relieving symptoms of BPH, has high acceptability and fewer side effects, and may reduce the use of other medications, thereby reducing risks and costs. The protocol of this systematic review and meta-analysis study aims to assess the efficacy and safety of moxa in the treatment of BPH. It also integrates the most recent and comprehensive clinical evidence in the field, with the hope of providing a wider variety of treatment options for patients with BPH and inspiring more peer specialists and physicians to conduct as many relevant studies as possible in the future.

## Author contributions

**Data curation:** Jiaze Wang.

**Formal analysis:** Tingting Deng, Xiaolu Sun.

**Methodology:** Hao Sun, Yuwei You, Ying Wang.

**Project administration:** Yuxia Ma.

**Resources:** Tingting Deng.

**Software:** Hao Sun, Qi Xun.

**Visualization:** Xiaolu Sun.

**Writing – original draft:** Jiaze Wang, Yuxia Ma.

**Writing – review & editing:** Jiaze Wang.
